# The PRO‐HOME Project. A multicomponent intervention for the protected discharge from the hospital of multimorbid and polytreated older individuals by using innovative technologies: A pilot study

**DOI:** 10.1111/hex.13872

**Published:** 2023-10-27

**Authors:** Alberto Pilotto, Erica Volta, Marina Barbagelata, Alessandra Argusti, Antonio Camurri, Niccolò Casiddu, Carlo Berutti‐Bergotto, Carlo Custodero, Alberto Cella

**Affiliations:** ^1^ Department Geriatric Care, Orthogeriatrics and Rehabilitation E.O. Galliera Hospital Genova Italy; ^2^ Department of Interdisciplinary Medicine “Aldo Moro”, University of Bari Bari Italy; ^3^ Department of Informatics, Bioengineering, Robotics and Systems' Engineering (DIBRIS) University of Genova Genova Italy; ^4^ Scientific Coordination Office, E.O. Galliera Hospital Genova Italy; ^5^ Department of Architecture and Design (DAD) University of Genova Genova Italy; ^6^ Informatic and Technology Unit E.O. Galliera Hospital Genova Italy

**Keywords:** aging, domotics, gerontechnology, multicomponent intervention, Multidimensional Prognostic Index, PRO‐HOME, robotics

## Abstract

**Backgroud:**

Discharge planning from the hospital of frail older patients is an important step to avoid inappropriate long‐stay hospitalizations and to prevent the risks related to the prolonged hospitalization. In this frame, we developed an experimental trial—‘PRO‐HOME’, a multicomponent programme of interventions for multimorbid and polytreated hospitalized older patients.

**Aim:**

The main aim of the study was to develop a protected discharge facility using a mini apartment equipped with advanced architectural and technological components to reduce the length of hospital stay of older participants (aged 65+ years old) admitted to the hospital for an acute event, deemed stable and dischargeable.

**Materials and Methods:**

This is a pilot randomized controlled study, comparing 30 hospitalized participants included in a multidimensional, transitional care programme based on information and communication technologies to 30 patients in standard usual care until hospital discharge.

**Results:**

We presented the study design of the PRO‐HOME programme, including architectural and technological components, the enrolment procedures, the components of the intervention that is physical activity, cognitive training and life‐style education and the evaluation method of the intervention based on the Comprehensive Geriatric Assessment to explore the changes in the individual domains that are target of the multicomponent intervention.

**Conclusions:**

The final results will suggest whether the PRO‐HOME programme represents a useful and feasible intervention to reduce the length of hospital stay of multimorbid and polytreated hospitalized older patients and improve their physical and cognitive performances and overall quality of life.

**Patient or Public Contribution:**

Due to the characteristics of the population of interest of the PRO‐HOME study, we involved in the study design and programme of the activities the participants enrolled in a previous smart home‐based project named MoDiPro carried‐out during a 3‐year period. The elderly participants from the local population involved were asked, by means of focus groups, for feedback on their experience in MoDiPro, and their suggestions were integrated into the design phase of the current PRO‐HOME project. The focus groups included open group interviews with a qualitative collection of the patients' feedback so that the participants could interact with each other.

## 1 INTRODUCTION

Prolonged hospital stay is associated with an increased risk of disability and iatrogenic diseases and an overall worsening of the multidimensional frailty condition and overall prognosis of older patients.^1^ Epidemiological studies suggest that about 8% of hospitalized older patients experienced prolonged hospital stay despite being clinically stable and fit for discharge with no need for further diagnostic or therapeutic procedures.^2^ The main reasons leading to delayed hospital discharge are often represented by the new‐onset disabilities and the complexity of older patients which needs further monitoring of the patients after the resolution of the acute event.^3^ In this context, recent studies suggest that developing multicomponent models of healthcare management for older multimorbid adults including care transition and continuity of care experiences might improve the activities of daily living (ADL) and the quality of life (QoL) and potentially reduce the length of hospital stay.^4^ However, the evidence is still mixed and the effectiveness of reducing hospital utilization uncertainty may be due to heterogeneity of treatment plans and poor adherence.^5^, ^6^

In the present paper, we describe the PRO‐HOME project, an innovative model of protected discharge programme for hospitalized older participants, developed through an interdisciplinary approach based on architectural, information technology and domotic and robotic components for clinical monitoring and developing cognitive and functional improvement protocols. Recent meta‐analysis and systematic review suggested that multicomponent intervention based on Comprehensive Geriatric Assessment (CGA) including physical activity programme, cognitive training, life‐style education and clinical diagnostic and therapeutic monitoring of the diseases are effective to improve functional and cognitive conditions and health status in the hospital setting.^7^, ^8^ The present study is conducted after the discharge from the Acute Geriatric Unit in a novel transitional care setting in which the management of patients is in charge of the hospital ward's healthcare staff in collaboration with the outpatient healthcare professionals to support clinical evaluations and interventions. In this new context, the role of gerontechnology, that is home automation and robotics, might be promising. Previous data of our group suggested the feasibility and usefulness of a smart posthospitalization facility for older people by using domotics, robotics and automated telemonitoring for a continuous assessment of frailty in hospitalized older people.^9^, ^10^ The novelty of the present study is to implement a tech‐focused setting that can facilitate hospital discharge to minimize unnecessary and potentially harmful prolonged length of stay (LOS) through CGA‐focused interventions that incorporate gerontechnology principles. This approach could change practice, challenge stigma around how older adults interact with new technologies and support CGA‐focused intervention strategies in the transitional care.

The main objective of the project is to develop and assess the effectiveness, in terms of reduction of length of hospital stay, of a protected discharge model for multimorbid and polytreated hospitalized older participants within a protected area designed and developed with advanced architectural and technological tools including domotics, robotics and telecare devices.

## 2 MATERIALS AND METHODS

### 2.1 Study design and inclusion criteria

The PRO‐HOME is a pilot, randomized controlled, no‐profit study cofunded by the Italian Ministry of Health and Liguria region in the frame of the Research Net‐Programme MULTIPLAT_AGE including four Italian regions, that is, Liguria, Campania, Calabria, Piemonte. Participants deemed eligible after having signed the informed consent are randomized into two arms (1:1 ratio), that is Group 1: standard care (control group) and Group 2: multicomponent intervention in the PRO‐HOME facility (intervention group). Multimorbid and polytreated patients are enroled in an Acute Geriatric Unit of a general hospital over the period of 1 year, started in May 2021. Geriatricians of the medical staff are responsible for the recruitment according to the inclusion criteria: once the patient signed the informed consent, the research team informs the medical staff if it will be included in Group 1 or 2, according to a computer‐generated randomization list. Inclusion criteria are: (1) participants aged 65 years or older; (2) admitted to the acute geriatric units for an acute event; (3) deemed stable and dischargeable from the hospital; (4) good personal autonomy in the ADL (according to the Katz ADL scoring ≥ 3/6) and in the instrumental‐ADL (according to the Lowton and Brody instrumental activities of daily living [IADL] scoring ≥ 6/8)^11^, ^12^; (5) normal cognition or mild cognitive impairment (Short Portable Mental Status Questionnaire [SPMSQ] ≤ 5/10)^13^; (6) who gave their signed informed consent. Participants with moderate or severe impairments in personal autonomy or cognitive and mobility functions, or with behavioural disorders or psychotic symptoms are not considered suitable to participate in the study. Patients included in Group 1 (control group) remain in the acute ward until the discharge (at home or in postacute facilities) following the standard hospital care in which patients are monitored and clinically followed‐up by the geriatric team according to a CGA‐based personalized clinical programme including physical and occupational therapy inside a hospital environment with all the related restrictions and rules, that is, limited visiting time slots by caregivers; patients included in the Group 2 (intervention group) are transferred to the protected area PRO‐HOME simulating a home context, eventually with a caregiver, and undergo the transitional care management with clinical and functional monitoring through:

1. Fixed and wearable sensors integrated in an automated monitoring system to measure vital parameters, motor activity and functional characteristics;
2. telepresence/teleassistance devices, also aimed at functional stimulation and environmental and personal safety;
3. robotic rehabilitation to reduce the risk of sarcopenia and falls.

For the participants included in the intervention group, the geriatrician responsible for the patient during the hospitalization provides indications about any residual healthcare needs to the health personnel involved in the trial to allow the planning of healthcare interventions during the period of stay in the protected area.

All participants will be followed‐up through a standardized telephone call planned at 1, 3 and 6 months after the hospital discharge to investigate any differences in health outcomes through a CGA‐based evaluation in the two different groups of participants.

### 2.2 The ‘PRO‐HOME eco‐system’

**Figure 1:**
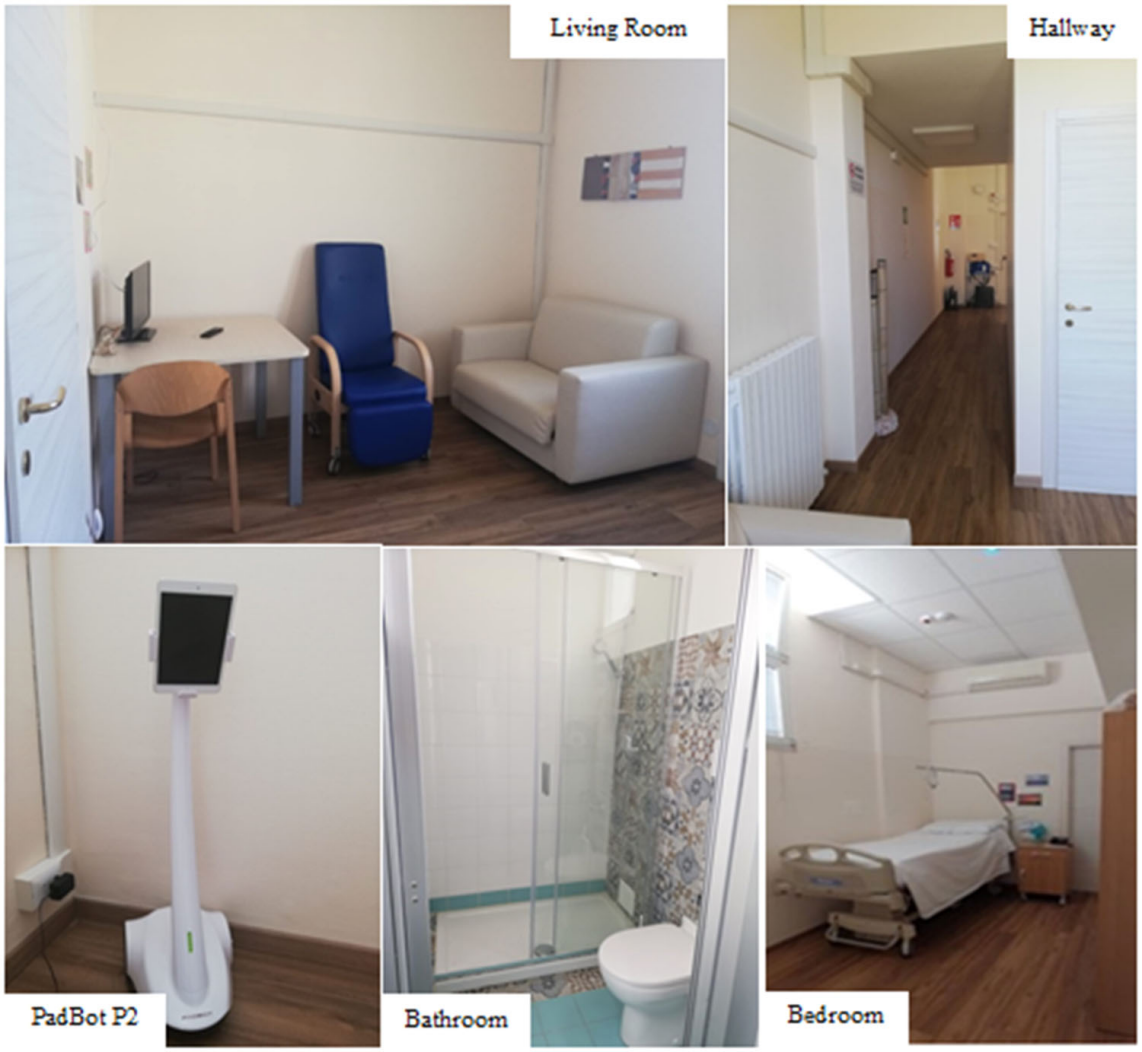
Some snapshots of the apartment realized for the PRO‐HOME protected discharge programme. It comprises a living room, with a bed‐sofa for hosting a caregiver and a bedroom for the patient. The walls and the sofa are bright with cream colours. There is also a bathroom fully equipped. In the hallway, a Microsoft Kinect Azure is mounted to monitor the quantity and quality of a patient's walk.

This project is based on the principle that by spending a period of transition in an environment with eminently domestic perceptual characteristics, people can regain the ability to live actively at home, gradually increasing their autonomy and mobility. Architectural choices have been conceived to ensure maximum safety, comfort and feasibility for patients, caregivers and healthcare professionals. The PRO‐HOME ‘ecosystem’ was designed and developed with materials, furniture, accessories and technological platforms able to monitor the protected area, facilitating communication between researchers, healthcare professionals, patients and their caregivers. The integration of ‘passive’ solutions (e.g., wall colour, room furnishings) and high‐tech systems create a domotic environment to ensure the well‐being and safety of patients and their caregivers. This home‐like ecosystem (Figure 1) consists of two rooms, that is, sleeping area and living area, plus a bathroom set up inside the facility and is equipped with an integrated technological system as detailed below. To guarantee the safety of the patient an integrated system of video/call alarms and communication 24 h/day between the guests of the apartment and the healthcare professionals (nurses and doctors) has been installed in the nurse room of the Geriatrics Unit as well in the PRO‐HOME apartment. Specifically, we provided (a) a telephone with prerecorded numbers in the bedroom, (b) a remotely monitoring system both in the bedroom and in the living room with two CCTV cameras, (c) a PadBot P2, a robot carrying a tablet and able to perform videocalls from/to the PRO‐HOME facility and (d) a lifesaver tool with an emergency call button in real time to guarantee ‘on demand’ visits by the medical staff (nurses and/or geriatricians) of the Acute Geriatrics Unit.

Healthcare professionals of the Geriatric Unit guarantee immediate intervention in case of emergency situations according to a specific and detailed operational emergency protocol developed by the researcher staff and approved by the hospital authorities.

In addition, caregivers have the opportunity to visit and stay inside the PRO‐HOME together with the patient during all the day and the night, while in a normal hospitalization their visits are limited and confined to specific time slots, also in relation to the recent Covid‐19 emergency.

**Figure 2:**
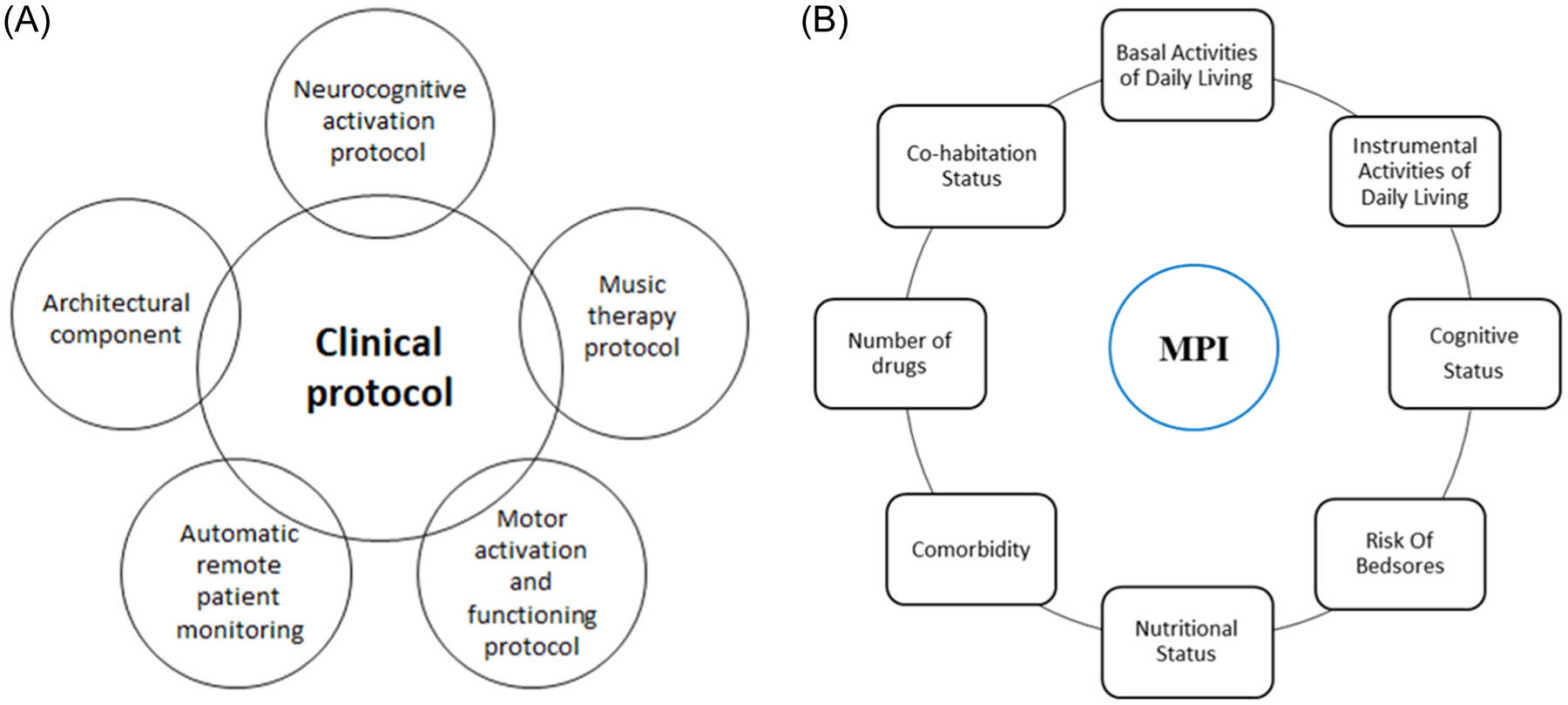
(A) The multicomponent intervention framework of the PRO‐HOME study protocol, and (B) the conceptual framework of the Multidimensional Prognostic Index (MPI) with the related eight domains.

The PRO‐HOME project reflects a multidisciplinary approach, involving architects, designers, software engineers and computer scientists for the design and technological equipment of the facility and health professionals such as geriatricians, physiotherapists, psychologists and nurses for the delivery of the clinical interventions. During the period spent in the apartment, the patient will be occupied in a series of neurocognitive and physical activation protocols, including an adapted music therapy relaxation protocol.^14^ In Figure 2A we showed an overall schematic view of the ecosystem technological assessment, including the physical, cognitive and music therapy protocols as detailed in the next paragraphs.

### 2.3 Technological set‐up for the ‘PRO‐HOME ecosystem’

The high‐tech integrated solutions include: (a) an infrared (IR) motion‐tracking camera (Kinect Azure from Microsoft) in the corridor to monitor gait speed; (b) a smartwatch (Fitbit Sense) for continuous monitoring of vital parameters and sleep monitoring; (c) one PadBot P2 for remote videocalls; (d) two CCTV cameras for remote monitoring in real time of the patient in the living and bedroom for security check and without recording images (every day and night functioning); (e) a 24 h/day lifesaver device with an emergency call button directly to the healthcare staff.

**Figure 3:**
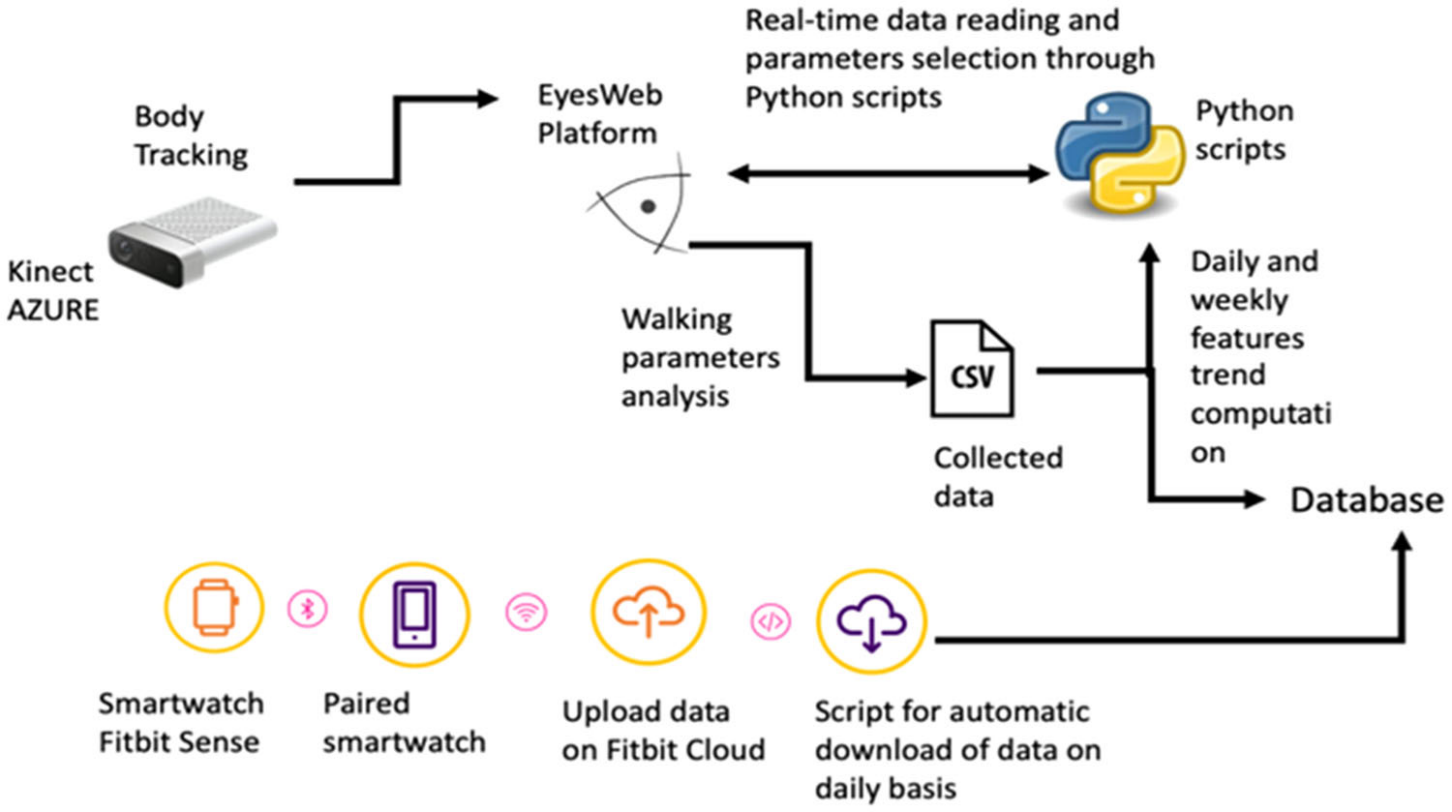
Schematic overview of the real‐time system for monitoring patient's clinical and functional parameters in the PRO‐HOME apartment.

All these devices guarantee the patient to move freely without restriction (via wireless communication), allowing analysis to be carried out on the data acquired: this implies the transmission of relevant technological data to a remote workstation running appropriate management software and the implementation of mathematical and statistical methods of analysis (Figure 3). Fitbit smartwatch and Kinect Azure camera are integrated in a real‐time system of monitoring of patient's clinical and functional parameters. In detail, the data captured through the IR camera are processed by a software framework, including several interconnected modules designed to perform different kinematic computations to detect participant movements. The collected data are continuously stored in databases accessible with a remote workstation and browsable using Python scripts that allow advanced search and filtering, providing daily and weekly trends. The framework was built using the EyesWeb XMI environment, an open software developed and widely used by the partners of the PRO‐HOME project^15^ and is controlled by a metalevel, scripted in Python language, that can activate the recording process when the camera detect motion and edit the parameters. Moreover, the meta‐level monitors the state of the recording and can reject spurious measurements like fleeting passages in the camera's cone of vision but not inside the corridor. During the phase that precedes the statistical analysis, this process allows the conservation of clean data that also undergo a manual screening with the aim of selecting the complete ‘walks’ over the entire length of the corridor and the measurements—both from the Kinect Azure and the Fitbit Sense smartwatch—recorded on the actual dates of stay in the PRO‐HOME apartment. All the measurements are stored merely for scientific purposes.

This device set will be able to provide the location of the guest within the indoor living environment, monitor his/her posture and movements, as well as the basal and ADL, IADL. For example, it will be intended to monitor the time the patient spends sitting in the armchair in the living area; the time, walking speed and stability in the upright posture during the passages through the corridor; the physical activity performed independently during the day; parameters related to the quality of sleep detectable by smartwatch.

During the stay in the PRO‐HOME protected area (expected average duration 5 ± 2 days) the following parameters will be collected for future analysis: (i) vital parameters through the smartwatch: blood pressure (daily and weekly mean values), heart and respiratory rates (daily mean values), oxygen saturation level (daily mean values); (ii) automated gait analysis through the Kinect Azure camera on a 4‐m linear path (i.e., the apartment corridor), including daily gait speed mean values (m/s) daily minimum and maximum gait speed and number of steps, daily average degree of body sway and lean in (grades)^16^; (iii) falls displayed in real‐time through the CCW cameras; (iv) spontaneous physical performance (i.e., average number of activity/inactivity hours) though the smartwatch; (v) duration (i.e., number of awakenings and time duration of light/deep/Rapid Eye Movement‐sleep) and quality of sleep thanks to the smartwatch.

### 2.4 Patient safety

The safety of the patient inside the PRO‐HOME is an important topic of the study project. This included 24‐h video cameras and a life‐saving device with an emergency button directly connected to the alarm system located in the geriatrics unit. As soon as the alarm system is activated and/or healthcare staff visually suspect/detect some emergency event inside the PRO‐HOME apartment, the procedures of the operational emergency protocol are carried out by the involved professionals.

Moreover, an emergency telephone line and a PadBot P2 are established for video‐calls from the patients to the medical staff of the unit and conversely to guarantee a primary clinical support in case of minor emergencies.

Nurses and geriatricians usually visit and check health parameters twice per day during the entire period of patient's stay in PRO‐HOME.

Concerning the collected health data, number of steps, heart rate, oxygen saturation level and quality of sleep are stored through the FitBit smartwatch, while quality of gait (i.e., body sway and lean in) and fluidity of movement are captured by the IR motion‐tracking camera: all these parameters will be analyzed by the medical and researching staff merely for scientific purposes.

### 2.5 The PRO‐HOME intervention protocols

The patients included in the PRO‐HOME intervention group are involved in a daily series of interventions of cognitive and physical activation, an educational programme aimed at enhancing a good care habits and a relaxation protocol of music therapy. All these activities are performed both inside the PRO‐HOME apartment and within specific outpatient clinics located within the hospital setting thanks to an existing collaboration. The cognitive intervention aims to stimulate all cognitive domains such as memory, language, attention and executive functions in a session lasting about 1 h/day.^17^ The educational programme includes daily sessions lasting 45 min of training in self‐management of chronic conditions,^18^ instructions on healthy diet following the principle of the Mediterranean diet, education to improve the sleep quality, information on the appropriate use of medications, body hygiene care.^19^

The physical‐functional activation protocol includes a personalized analysis of the patient's functional performance assessed by clinical parameters, and a robotic evaluation carried‐out by the Hunova robot with the support of a physical therapist to study static and dynamic equilibrium performances, reactive balance and the sit‐to‐stand tasks.^20^, ^21^, ^22^ The rehabilitation programme lasts about 1 h/day and aims to intervene on dysfunctions of the musculoskeletal system, for example, joint instability, postural alterations, alterations in the kinematics of movement that in turn increase the risk of further functional deterioration, falls and/or limitations in social relations.

Finally, the patient is trained on the use of a music‐therapy self‐administered protocol, conceived for relaxation and well‐being. The protocol, initially applied in healthcare fields such as work‐related stress^23^ and telemedicine is based on a series of algorithmic music compositions, created ad hoc for stress and pain relief.^22^ In the PRO‐HOME ecosystem, therapeutic music listening is conceived to be a free activity that the patient can choose if adopted during the free time spent in the apartment. The therapeutic music listening protocol is then evaluated through an adapted version of Client Satisfaction Questionnaire 8 (CSQ‐8)^24^ and a usability test to check whether the device used (e.g., the tablet) is perceived as an obstacle by the patient.

### 2.6 Clinical and multidimensional assessment

At baseline, in all participants demographic information, that is age, gender, marital status, level of education and clinical data, that is blood pressure, heart rate and oxygen saturation level are collected, additionally to the principle and secondary diagnoses and treatments in progress.

All patients from both the Intervention and the control group undergo a standard CGA that includes functional status (ADL, IADL),^11^, ^12^ cognitive status by the SPMSQ,^13^ risk of bedsores measured by the Exton‐Smith scale,^25^ nutritional status by the Mini Nutritional Assessment‐short form,^26^ comorbidity by means of the Cumulative Illness Rating Scale,^27^ the number of drugs taken by the patient and the cohabitation status of the subject (alone, in institution, with his/her family). From these eight domains is calculated the Multidimensional Prognostic Index (MPI), a numerical prognostic index of negative outcomes (mortality, institutionalization, hospitalization) developed and validated in hospitalized older people (Figure 2B).^28^ As a predictor of negative clinical outcomes, the MPI is one of the most used instruments to identify and measure the multidimensional frailty in older people.^29^ The MPI can be expressed both as a continuous numerical index from 0 (no risk) to 1 (maximum risk) or in three degrees of risk: low (MPI‐1), moderate (MPI‐2) or severe (MPI‐3) according to appropriate cut‐offs (MPI‐1 ≤ 0.33; MPI‐2 between 0.34 and 0.66; MPI ≥ 0.67).^28^

Moreover, all participants undergo the Geriatric Depression Scale short form (GDS‐15)^30^ and the quality‐of‐life test (QoL SF‐12).^31^ The GDS‐15 is a widely used rating scale for the detection of depressive symptoms in older people. It consists of 15 binary items (Yes/No) scale to detect depressive symptoms: the final score ranges from 0 (no signs of depressive symptoms) to 15 (maximum severity of depression). The standard version of the QoL SF‐12 consists of 12 items that explores physical and mental health. It consists of four scales, each including two items, to measure physical functioning, physical health, emotional state, mental health and four further scales to measure physical pain, vitality, social activities and general health. The scale is designed to compose two indices, that is, the physical component summary/physical health and the mental component summary/mental health. The person is asked to answer about how he/she feels and how well he/she can perform daily activities based on the date of assessment and the previous 4 weeks.

Compared to the control group (Group 1) which are administered a CGA evaluation, the participants included in the Group 2 (intervention) a further series of clinical and functional parameters are monitored by health professionals including: (a) the hand grip test; (b) the short physical performance battery (SPPB),^32^ that is a widely used physical performance test including three different sections to measure (i) assessment of balance in three tests; (ii) the gait speed on 4 linear metres; (iii) the ability to perform, for five consecutive times, sit‐to‐stand from a chair without using the upper limbs. The total SPPB scale score ranges from 0 to 12 points. At the end of the period of staying in the PRO‐HOME facility a CSQ‐8^24^ is also carried out in all participants. During the stay in the PRO‐HOME apartment, drug therapy, that is, adherence to the therapeutic plan, and nursing care interventions including nursing care, number and type of diagnostic tests or consultations and the emergency health interventions are monitored and recorded. All data are collected by the healthcare staff, including specialized physicians, geriatricians, nurses and physical therapists for the related sections in a paper format from both the geriatric ward and outpatient clinics: this procedure lasts about half an hour. Then, the researcher team will enter all data in a specific platform developed within the project.

During the follow‐up evaluations (1, 3 and 6 months after the discharges from the hospital), all participants will be contacted by a standardized telephone call in which the healthcare staff of the hospital will administer: (a) the telephone version of the MPI (TELE‐MPI),^33^ (b) QoL (SF‐12), (c) changes in the drug‐therapy, (d) number of other hospitalizations or institutionalizations.

### 2.7 Statistical analysis

The primary outcome, that is LOS, will be tested by means of a *t*‐test for independent data. Multivariate linear regression models will be used to assess the LOS differences between the two groups (intervention vs. control groups) with respect to the clinical and functional data and adjusting it for demographic and all other possible confounders. Descriptive analyses will be performed using means (*M*) and standard deviations (SDs) for age, gender and years of education. Secondary outcomes will be analyzed with: *M*, SDs for continuous variables (i.e., vital parameters, gait speed, physical parameters, MPI, sleep parameters, QoL, level of satisfaction, depression parameters). Frequencies and percentages will be used for the analyses of categorical values (i.e., MPI categorical domains, rate of rehospitalization, institutionalization and mortality). To compare data between the two groups, the independent *t*‐test (or Mann–Whitney *U* test for data not normally distributed) and the *χ* ^2^ test will be used for continuous variables and categorical, respectively.

### 2.8 Sample size calculation

To the best of our knowledge, no reliable data are available in the literature on the assessment methods of a protected discharge programme such as that described and tested in the PRO‐HOME project. Some published data indicate that a mean hospital stay (primary endpoint) of approximately 16 days (SD = 4) can be expected in the target population over a period of 6 months. Assuming a two‐tailed *α* error of 5%, a sample size including 60 randomized participants (30 participants in the Group 1 and 30 participants in the Group 2) the study has a power of 95% in finding a difference between the two arms of 4 days, that is *M* = 12 days in the intervention arm versus 16 days in the control arm; the study shows a power of 80% in finding a difference between the two arms of 3 days (i.e. mean 13 days vs. 16 days). The calculations considered a lost‐to‐follow‐up rate of 10%. Based on these considerations, the number of participants to be included in the study was set at 60 persons, that is, 30 for each of the two study groups. To prevent any drop‐out of participants, geriatricians of the Geriatric Unit explain in detail the innovation of participating in the PRO‐HOME project with its intervention protocols, so that patients know what they are getting into, without unexpected issues.

### 2.9 Privacy and ethical issues

The MULTIPLAT_AGE project has been approved by the Italian Ministry of Health (No. 44761 dated 27 February 2018) and by the Ethical Committee of Liguria Region on 26 March 2018; the PRO‐HOME project has been approved by the Ethical Committee of Liguria Region (register No. 176/2021) on date 17 May 2021. All patients signed an informed consent. To guarantee respect for the patient's privacy, it is not foreseen to record camera images that make the enroled participant recognizable: the stored data are not able to directly identify patients. All the clinical data and the parameters recorded by sensors and technological devices are stored and secured in the server database of the Galliera Hospital. Access to these data is allowed only to research partners via remote desktop.

## 3 EXPECTED RESULTS

The PRO‐HOME project was designed to develop and validate a protected discharge plan to reduce the length of hospital stay as primary aim by activating a protected discharge programme for multimorbid and multitreated hospitalized older participants. The project aims at validating and exploiting a multidisciplinary approach, in which clinical as well as motor, cognitive and relaxing activities are integrated in a comprehensive caring approach by using architectural and technological components to guarantee a continuous monitoring of clinical, functional and safety parameters.

Secondary objectives include the short‐ and long‐term evaluation of: (a) the effectiveness of technological monitoring of functional and psychological status, including ADL, IADL, degree of multidimensional frailty as assessed by the MPI, gait speed, vital parameters and sleep quality monitoring compared to the control group; (b) the acceptability, satisfaction and QoL of patients included in the PRO‐HOME programme compared to controls; (c) the potential negative events collected during the follow‐up such as falls, rehospitalization, institutionalization, mortality during a 6‐month follow‐up period.

## 4 DISCUSSION

The main significance of this project is to develop and validate an innovative health‐care model able to promote appropriate and tailored interventions to multimorbid and polytreated older participants who are at risk of inappropriate or unnecessary prolonged hospital stay and to guarantee a favourable cost‐effective ratio for the older participants and the National Health System. The PRO‐HOME framework is based on a deep literature review, particularly considering previous experiences.^10^, ^34^

The model is innovative due to: (a) the wide use of high‐tech systems able to provide data that are processed by advanced data analysis tools to ensure the daily average data collection of clinical and functional parameters including automated evaluation of risk of falls, inappropriate drug use or the occurrence of functional and cognitive impairments; (b) the architectural and technological ‘older‐friendly’ facilities; (c) an interdisciplinary and integrated approach to older adults well‐being through a patient‐tailored motor, cognitive and music relaxation protocol; (d) a great attention paid to the personalization of interventions to guarantee a high grade of satisfaction and QoL; (e) an improved and integrated use of health resources; (f) a multicomponent intervention including education, physical and cognitive activation and a music therapy intervention, in older people in the hospital environment.

To the best of our knowledge this integrated approach is a unicum so far, while in literature there are several findings related to low‐cost technology deployment in smart home for elderly,^10^, ^34^ none of them consider a deeper understanding of participants' frailty, the architectural components, the self‐care, and activities that can be carried out in the same protected environment. Nevertheless, basing the technological ecosystem on low‐cost and commercial technologies allow the PRO‐HOME model to be easily translated and disseminated in real practice.

We excluded participants with moderate and severe functional and cognitive impairments, including Parkinson's disease. For instance, Fitbit has been shown to have higher error in individuals with Parkinson's disease.^35^

However, future studies are needed to investigate and deepen the following unexplored aspects: (i) perception of technology by older people, (ii) potential limitations in accuracy of technological devices in patients with mobility disorders,^35^ (iii) degree of satisfaction about the PRO‐HOME experience through open‐ended questions, (iv) possible negative effects of a long hospitalization as secondary outcomes.^3^

## 5 CONCLUSION

In this paper we presented both clinical and technological components of a prototype of discharge plan from the hospital to home, called PRO‐HOME for hospitalized older multimorbid and poly‐treated patients. This model applies the tenets of multidimensional assessment and management of a hospital discharge condition (i.e., educational advice, rehabilitation programme, relaxation techniques) using also technological, domotic and robotic solutions, installed in a two‐room apartment inside the hospital, to track and monitor the safety and the clinical parameters of the patients.

## PRO‐HOME PROJECT INVESTIGATORS

Daniela Cademartori, Marco De Benedetto, Alberto Ferri, Simone Ghisio, Mauro Nelli, Silvia Pericu, Claudia Porfirione, Camilla Prete, Sanket Sabharwal, Barbara Senesi, Federica Solari, Francesco Vallone, Simonetta Galliani

## AUTHOR CONTRIBUTIONS

Alberto Pilotto and Alberto Cella conceived the PRO‐HOME project and the clinical study; they are responsible for the management of the project and contribute to write the manuscript. Erica Volta contributed to the design of technological setup, developed the music therapy protocol, and contributed to write the manuscript. Antonio Camurri, Simone Ghisio and Sanket Sabharwal developed the technological design, framework and set‐up of the PRO‐HOME; Niccolò Casiddu, Silvia Pericu, Claudia Porfirione and Federica Solari developed the architectural design and framework of the PRO‐HOME ambients; Carlo Berutti‐Bergotto and Marco De Benedetto contribute to the informatics development of the PRO‐HOME; Mauro Nelli, Alessandra Argusti, Daniela Cademartori and Alberto Ferri are responsible for the management of the PRO‐HOME project; Francesco Vallone, Camilla Prete, Barbara Senesi, Marina Barbagelata, Carlo Custodero and Simonetta Galliani contributes to the clinical protocol design and developed the neurocognitive and rehabilitation programmes. All the co‐authors contribute to review the manuscript and approved the final draft submitted.

## CONFLICT OF INTEREST STATEMENT

The authors declare no conflict of interest.

## Data Availability

Data sharing is not applicable to this article as no datasets were analyzed during the current study.
